# Meeting of Clergy at the North-Eastern Hospital for Children, Hackney Road, E.

**Published:** 1904-11-26

**Authors:** 


### MEETING OF CLERGY AT THE NORTH-EASTERN HOSPITAL FOR CHILDREN, HACKNEY ROAD, E.

On Thursday, the 17th inst., a meeting of clergy was held at the North-Eastern Hospital for Children to consider the critical financial position of the hospital. The hon. chaplain, the reverend W. G. Morcom, presided, and among those present were Lord William Cecil (Chairman of the Hospital Committee), the Hon. the Reverend A. G. Lawley (Rector of Hackney), the Reverend H. V. S. Eck (Rector of Bethnal Green), Mr. Wright Adcocks, and Mr. C. G. J. Port.

The districts surrounding the hospital are among the poorest and most thickly populated in London. Within a mile radius of the hospital there is a population of over 361,000, about half of which consists of children under twelve years of age. The hospital has now a debt of £9,000, part of which is the cost of the new building; but £4,000 has been incurred by the increased work of the institution. Unless funds are swiftly forthcoming, it will be necessary to close half the wards at the end of December.

The Chairman strongly urged the needs of the hospital. Many families, he said, in the neighbourhood lived in one room only, and this fact, coupled with the poverty of the people, rendered the children's hospital a dire necessity. If the fifty-seven beds were closed it would affect 1,000 patients. When the closing of the beds was suggested there had been almost a panic in the neighbourhood, and £8 in coppers had been deposited in the collecting-boxes at the door. The Hospital Committee had come to the end of their borrowing resources, and they appealed to the clergy in the wealthier parts of London to help them in their present difficulty.

The Reverend H. V. S. Eck proposed the following resolution, which were carried unanimously: "That this meeting of the clergy of all denominations of East and North-East London strongly urges upon the public the vital importance to the people of these crowded districts of keeping open all the wards of the North-Eastern Hospital for Children, and very earnestly appeals to every responsible man and woman to contribute towards the sum of £4,000 required before the end of the year to enable the disastrous step of closing 57 of the beds to be avoided." And "That in view of the serious danger in which the North-Eastern Hospital for Children stands of being compelled at the end of December to close half its wards, for want of funds to carry on its work amongst the children of the sick poor, this meeting of the clergy from the surrounding crowded districts unanimously undertakes to use every endeavour to arrange special offertories, or collections, in aid of the institution before the end of the year."

